# Response to letter to the editor regarding “posterior intra-articular fixation stabilizes both primary and secondary sacroiliac joints: a cadaveric study and comparison to lateral trans-articular fixation literature.”

**DOI:** 10.1186/s13018-023-04080-1

**Published:** 2023-08-09

**Authors:** Dawood Sayed, Kasra Amirdelfan, Corey Hunter, Oluwatodimu Richard Raji

**Affiliations:** 1 The University of Kansas Medical Center, Kansas City, KS USA; 2 IPM Medical Group, Walnut Creek, CA USA; 3 Ainsworth Institute of Pain Management, New York, NY USA; 4 Medical Device Development, 2390 Mission Street, Suite 8, San Francisco, CA 94110 USA

**Dear editor,**

We appreciate the opportunity to respond to the letter by the authors of Lindsey et al. [1] regarding our recent article Sayed et al. [2]. The letter aims to call the comparative results into question, by presenting concerns predominantly with regard to statistical parameters. While we thank the authors for their review of our recent article, the concerns presented by the letter, in most cases, are not supported by objective evidence and are at best inaccurate portrayals of the background, aims, materials, and methodology indicated in Sayed et al. [2]. Our responses to each comment are noted below.

## Specimen demographics

The authors Lindsey et al. [1] state that the mean specimen age differs between Sayed et al. [2] and Lindsey et al. [1], and thus are not comparable. However, a closer review of the specimen demographic from Lindsey et al. [1], shows that specimens as young as 28 and 36 years were utilized. Thus, the specimens aged 34–37 years utilized in Sayed et al. [2], fall within the demographic evaluated by Lindsey et al. [1]. To further address any questions or concerns regarding specimen age, we would request that the authors of Lindsey et al. [1] provide the datasets for each specimen utilized, to enable a much closer one-to-one comparison between demographically identical specimens. These datasets, which have twice been previously requested, have not been made available by the authors (Lindsey et al. [1]) upon reasonable request.

The authors also state that there is a known relationship between increasing age and decreasing bone density. However, the letter does not cite any studies, nor the bone quality of the specimens utilized in Lindsey et al. [1] to support this claim. A brief review of recent publications with regards to the bone density of the sacrum offers more context which disproves this claim when taking into consideration the consistent implantation site which is located at the S2 region of the sacrum in Sayed et al. [2] (Figs. 2 and 5) and Sayed et al. [3] (Fig. 3).

Mand et al. [4] analyzed fifty-four sacral models segmented from routine computed tomography scans of patients aged 18 to 80 + years, to assess the impact of age and sex on sacral bone density. They reported that within the S2 region of the sacrum, which corresponds to the implantation site in Sayed et al. [2] and Sayed et al. [3], the cortical endplate and adjacent trabecular bone densities were consistent across all ages, as shown in Figs. 2 and 4 of Mand et al. [4].

Flanigan et al. [5] also reported that across forty patients aged 36–84 years, the bone density at the S2 sacral ala was consistently osteoporotic with significantly less bone density compared to the lumbar vertebral body at − 11.9 HU (S2) vs 111 HU (L1-4). Similar to Mand et al. [4], Flanigan et al. [5] also reported that the S2 sacral ala was unaffected by teriparatide treatment at − 8.43 HU (*p* = 0.54) in contrast to L1-4 at 130 HU (*p* < 0.001). These studies thus indicate that given the consistent location of the implantation site in the S2 region as shown in Figs. 2 and 5 of Sayed et al. [2] and Fig. 3 of Sayed et al. [3], the density of the bone adjoining the allograft, is most likely unaffected by the age of the specimens utilized.

However, for the S1 implantation sites utilized in the lateral trans-articular technique Lindsey et al. [1], bone density varies significantly with age (Mand et al. [4], Flanigan et al. [5]). Thus, it is more than likely that this technique would be significantly impacted by patient age as pertains to bone density. An analysis of the FDA’s Adverse event database performed by Rahl et al. [6], supports this postulation, as the ‘loss of osseointegration’ is the second most prevalent cause of adverse events such as undesired nerve stimulation, arthralgia, and bone fracture, with the lateral trans-articular technique.

## Sample size

The authors (Lindsey et al. [1]), state that the parameters for sample size calculations are not identical to Soriano-baron et al. [7] referenced in Sayed et al. [2]. The authors cite specifically the effect size of 50% versus 30%.

We would like to clarify that only the parameters of the standard deviation, significance level, and statistical power, were chosen from the referred article. As there had not been any reported biomechanical study of the intra-articular approach, the effect size was chosen based on the results obtained from our first and second specimens. In these specimens, unilateral fixation generated 72%, and 58% motion reduction, for an average of 65% reduction in flexion–extension. This effect size of 65% justifies the conservative selection of 50%, utilized in Sayed et al. [2]. This resulted in a sample size estimation of 4 specimens, however, two (2) additional specimens were utilized, for a total sample size of 6 specimens.

The authors (Lindsey et al. [1]), also state that sample size utilized is minimal, partly due to the quantification of specimens by the number of sacroiliac joints versus the number of pelvises. However, the analysis presented in Sayed et al. [2] and Lindsey et al. [1], are of sacroiliac joint stability and not overall pelvis stability. There are no studies yet, which adequately assess pelvis stability, given the complexity of pelvic loads. As both implants are indicated for sacroiliac joint fixation and arthrodesis, not pelvic stabilization, the quantification of specimens must be according to the number of sacroiliac joints.

## Data pooling

The authors (Lindsey et al. [1]), state that the pooling of the primary and secondary sacroiliac joints presents methodological and statistical issues. The letter, however, misrepresents the statistical basis for pooling, by utilizing a paired t-test of the left and right, instead of the primary and secondary sacroiliac joints to justify their claim. This misrepresentation assumes that only the left or only the right joints were selectively treated as the primary joint, which is a contrary to the methodology reported in Sayed et al. [2] which states “The primary joints (left/right) were chosen at random for each pelvis”.

While the minimum *p*-value. < 0.2 from a pair-wise analysis of the left and right joints does not strongly justify equivalence, further statistical analysis of the primary and secondary joints under intact, and unilateral conditions using paired t-test, yields p-values ranging from 0.37 to 0.70, and 0.62 to 0.70, respectively. Thus, using the statistical rationale provided by the authors Lindsey et al. [1] in the letter, pooling of the samples is hereby justified, as the primary and secondary joint are equivalent, in both the intact and unilateral treatment states.

## Implant placement

The authors Lindsey et al. [1], state that the use of varying trajectories invalidates the results and comparison presented in Sayed et al. [2], given that the study compares between “trajectories”. However, a brief review of Sayed et al. [2], shows that this claim is also a misrepresentation. It is clearly stated in Sayed et al. [2] that the study did not aim to compare between implant placement, instead the study aimed to compare between the fixation mechanisms/principles (intra-articular vs trans-articular), and between the surgical access/approach (posterior vs lateral). This was especially necessary, as the authors Lindsey et al. [1] did not report the implantation trajectory or location used for each specimen. It is however important to note that that while implantation trajectory Sayed et al. [2] varied, the actual location of the implantation site within the joint was consistent irrespective of the different clinicians who performed the implantation. This is clearly shown in Sayed et al. [2] (Figs. 2 and 5) and Sayed et al. [3] (Fig. 3).

The authors’ Lindsey et al. [1] concern is however understandable, when utilizing the lateral trans-articular approach, as while Soriano-baron et al. [7] did not note statistically significant differences, the mean effect size upon device placement was impacted by implant position. In addition, an analysis of the FDA's Maude database by Rahl et al. [6] supports the authors’ concern for the lateral trans-articular technique, as ‘device malpositioning’, is the most prevalent cause of adverse events with the lateral trans-articular technique, utilized in Lindsey et al. [1]. Given that this leads to adverse events such as undesired nerve stimulation, arthralgia, and bone fracture, the equivalent results in flexion–extension, and superior results in lateral bending and axial rotation, indicates consistent performance and minimized risk profile of the posterior intra-articular in comparison to the lateral trans-articular technique, across variations in trajectory.

## Clinical evidence

The authors Lindsey et al. [1], recommend a 2017 review by Dengler et al. [8] of only three (3) prospective trials, funded by the authors’ industry affiliation, and which investigates only the triangular rod technique, a sub-division of lateral trans-articular techniques, as the standard to be utilized in providing a safety overview for all lateral sacroiliac joint devices.

The letter also states that the introductory background in Sayed et al. [2], selectively references postoperative complication rates. However, a brief review of the introductory background shows that the more recent 2019 and 2020 publications of Shamrock et al. [9] and Martin et al. [10], respectively, were utilized. These are systematic and narrative review studies inclusive of all published variations of the lateral trans-articular techniques, such as twelve (86% of all reviewed publications) and seventeen (68% of all reviewed publications) reports on the triangular rod technique, respectively. These reviews at the minimum, included “7 retrospective single-center case series, 2 prospective multicenter randomized controlled trials, 2 prospective single-center case series, 1 prospective multicenter comparative cohort study, 1 retrospective multicenter case series, and 1 retrospective single-center comparative cohort study.” Shamrock et al. [9].

While popular, the triangular rod technique is not the only lateral trans-articular technique being utilized by clinicians. Therefore, the introductory background of Sayed et al. [2] provides an objective and unbiased assessment of the lateral trans-articular technique group, in the same way that it does for the posterior intra-articular technique group.

In summary, the authors Lindsey et al. [1] in the letter, fail to provide objective evidence, and in some cases, any evidence at all, such as bone density measurements, implant position, implant trajectory, or individual specimen ranges of motion, from Lindsey et al. [1], to support the concerns raised. This lack of objective data in addition to the methodological misconstructions identified, calls into question the purity of the motivation, and validity of the foundation upon which these claims are built; inversely contributing to a stronger overall impact of the results presented in Sayed et al. [2].

## Data Availability

Not applicable.
