# Correction: Response to letter to the editor regarding “posterior intra-articular fixation stabilizes both primary and secondary sacroiliac joints: a cadaveric study and comparison to lateral trans-articular fixation literature.”

**DOI:** 10.1186/s13018-023-04106-8

**Published:** 2023-08-31

**Authors:** Dawood Sayed, Kasra Amirdelfan, Corey Hunter, Oluwatodimu Richard Raji

**Affiliations:** 1 Medical Center, The University of Kansas, Kansas City, KS USA; 2 IPM Medical Group, Walnut Creek, CA USA; 3 Ainsworth Institute of Pain Management, New York, NY USA; 4 Medical Device Development, 2390 Mission Street, Suite 8, San Francisco, CA 94110 USA

**Correction: Journal of Orthopaedic Surgery and Research (2023) 18:590** 10.1186/s13018-023-04080-1

Following publication of the original article [1], the references 2 and 3 in the reference list needs to be interchanged.

Reference 2 should be the JOSR 2023 publication

Reference 3 should be the Med Evidence and Research 2021 publication

The original article has been corrected.
